# Severity of Influenza A 2009 (H1N1) Pneumonia Is Underestimated by Routine Prediction Rules. Results from a Prospective, Population-Based Study

**DOI:** 10.1371/journal.pone.0046816

**Published:** 2012-10-11

**Authors:** Agnar Bjarnason, Gudlaug Thorleifsdottir, Arthur Löve, Janus F. Gudnason, Hilmir Asgeirsson, Kristinn L. Hallgrimsson, Berglind S. Kristjansdottir, Gunnsteinn Haraldsson, Olafur Baldursson, Karl G. Kristinsson, Magnus Gottfredsson

**Affiliations:** 1 Faculty of Medicine, University of Iceland, Reykjavik, Iceland; 2 Department of Medicine, Landspitali University Hospital, Reykjavik, Iceland; 3 Department of Virology, Landspitali University Hospital, Reykjavik, Iceland; 4 Department of Emergency Medicine, Landspitali University Hospital, Reykjavik, Iceland; 5 Department of Clinical Microbiology, Landspitali University Hospital, Reykjavik, Iceland; National Institutes of Health, United States of America

## Abstract

**Background:**

Characteristics of patients with community-acquired pneumonia (CAP) due to pandemic influenza A 2009 (H1N1) have been inadequately compared to CAP caused by other respiratory pathogens. The performance of prediction rules for CAP during an epidemic with a new infectious agent are unknown.

**Methods:**

Prospective, population-based study from November 2008–November 2009, in centers representing 70% of hospital beds in Iceland. Patients admitted with CAP underwent evaluation and etiologic testing, including polymerase chain reaction (PCR) for influenza. Data on influenza-like illness in the community and overall hospital admissions were collected. Clinical and laboratory data, including pneumonia severity index (PSI) and CURB-65 of patients with CAP due to H1N1 were compared to those caused by other agents.

**Results:**

Of 338 consecutive and eligible patients 313 (93%) were enrolled. During the pandemic peak, influenza A 2009 (H1N1) patients constituted 38% of admissions due to CAP. These patients were younger, more dyspnoeic and more frequently reported hemoptysis. They had significantly lower severity scores than other patients with CAP (1.23 vs. 1.61, *P* = .02 for CURB-65, 2.05 vs. 2.87 for PSI, *P*<.001) and were more likely to require intensive care admission (41% vs. 5%, *P*<.001) and receive mechanical ventilation (14% vs. 2%, *P* = .01). Bacterial co-infection was detected in 23% of influenza A 2009 (H1N1) patients with CAP.

**Conclusions:**

Clinical characteristics of CAP caused by influenza A 2009 (H1N1) differ markedly from CAP caused by other etiologic agents. Commonly used CAP prediction rules often failed to predict admissions to intensive care or need for assisted ventilation in CAP caused by the influenza A 2009 (H1N1) virus, underscoring the importance of clinical acumen under these circumstances.

## Introduction

Influenza pandemics are associated with significant morbidity and mortality, mostly due to respiratory tract infections. The severity of the three pandemics of the 20^th^ century differed greatly, ranging from case fatality rate of less than 0.5% for the 1968 Hong Kong pandemic, to 3% during the Spanish flu [1]. Studies on lung tissue from victims of the Spanish flu of 1918 have confirmed the existence of primary viral pneumonia but also implicated bacterial infections, most notably due to *Streptococcus pneumoniae* [2]. Recent research shows that approximately one-third of patients with community-acquired pneumonia (CAP) requiring hospitalization have viral and bacterial co-infections, most commonly influenza and *S. pneumoniae* [3]. During non-pandemic influenza seasons the virus causes up to 8% of CAP cases warranting admission [3]. In order to improve clinical decision making and optimize utilization of resources in health care, clinical prediction rules and prognostic models of patients with CAP have been developed, most notably CURB, CURB-65, and pneumonia severity index (PSI) [4], [5]. These clinical tools have been validated and their use is advocated in clinical guidelines [6], [7]. However, the prediction rules were developed during an inter-pandemic influenza period and therefore may not be optimally suited to predict the clinical course in patients with CAP caused by novel infectious agents. In 2009 the World Health Organization (WHO) declared an influenza A (H1N1) pandemic, the first in over 40 years [8]. An increase in the rate of severe pneumonia and a shift in the age distribution was noted first in Mexico and subsequently in Australia [9], [10]. In contrast, data from Wisconsin suggested that the 2009 H1N1 infections were similar in severity to seasonal influenza [11], while a study from Singapore reported that when compared to seasonal flu the pandemic H1N1 virus caused milder symptoms [12]. Interestingly, however, the Wisconsin study reported a higher proportion of H1N1 infections resulting in pneumonia, compared with H3N2 infections [11], and Jain et al found pneumonia in 43% of pandemic influenza admissions [13]. These apparent contradictory findings could potentially be explained by different dominant viral subtypes in the seasonal influenza control groups, herd immunity and host genetics [14], but they could also be methodological, resulting in different selection of patients. During the 2009 influenza pandemic a prospective study on CAP was ongoing in Reykjavik, Iceland. The pandemic offered a unique opportunity to study the impact of the influenza A 2009 (H1N1) pandemic on hospital admissions due to pneumonia. The primary aim of the study was to examine and describe the symptoms, microbial etiology, treatment and outcomes of all patients requiring hospital admission due to CAP. The secondary aim of the study was to compare patients admitted with CAP due to influenza A 2009 H1N1 to patients infected by other etiologic agents. This comparison included clinical characteristics of the patients, including symptoms, results of laboratory studies and performance of the CURB-65 and PSI prediction rules.

## Methods

### Ethics statement

The study was approved by the Ethics Committee of Landspitali University Hospital and Data Protection Authority. All participants or proxy provided written informed consent.

### Setting and inclusion criteria

Iceland is a 103 000 km^2^ island with a 2008 mid-year population of 319 355. Landspitali University Hospital in the capital Reykjavik has 700 beds, constituting 70% of the national total; serves as the only secondary care hospital for more than 63% of the entire population and provides ICU care for over 90% of the country. All patients ≥18 years of age with newly diagnosed pneumonia requiring hospital admission from December 2008 through November 2009 were eligible for inclusion if they had a new infiltrate on a chest-X ray confirmed by a physician and fulfilled at least two of the following criteria: temperature <36°C or >38.3°C, diaphoresis, chills, chest pain, or new onset of cough or dyspnea [15]. Admissions were reviewed daily during the study period and all possible cases with triage diagnosis of pneumonia were approached for participation by at least one of the authors. Exclusion criteria were hospitalization within the preceding 14 days or significant immunosuppression (HIV infection, corticosteroid use exceeding 10 mg prednisolone daily, other immunosuppressive treatment or active cancer treatment) [15].

### Data and sample collection

Following inclusion, participants underwent a structured interview and examination. Sputum was collected for Gram-stain and culture, blood cultures were taken prior to antibiotic treatment and urine for pneumococcal and *Legionella* antigen testing (BinaxNow® *S. pneumoniae* and BinaxNow® *Legionella*; Inverness Medical Innovations). High-quality sputum was defined as previously described [16]. A throat swab was collected for polymerase chain reaction (PCR) testing. Results of other etiologic studies, initiated by the treating physicians were noted. All participants were assessed for Pneumonia Severity Index (PSI), CURB-65 and APACHE II scores [4], [5], [17]. The Icelandic National Registry was cross-checked to detect 30 day mortality in discharged patients. Data on number of admissions was provided by Landspitali University Hospital.

### PCR analysis for influenza and atypical bacteria

All available samples were stored at −80°C for analysis after the study period. DNA/RNA was extracted with QIAmp® DNA Blood Mini kit (QIAGEN®) and MagNA Pure Compact Nucleic Acid Isolation Kit I (Roche Diagnostics®). PCR analysis for influenza A H1N1 and atypical bacterial causes (*Mycoplasma pneumoniae, Chlamydophila pneumoniae* and *Legionella pneumophila*) was performed with the 7500 Fast Real-Time PCR System (Applied Biosystems™) using the Ambion® AgPath-ID™ One-Step RT-PCR Kit (Applied Biosystems™) as well as the appropriate primers (Sigma-Aldrich®) and probes (Applied Biosystems™). Primers and TaqMan-MGB probes for *M. pneumoniae, C. pneumoniae* and *L. pneumophila* detection were based on the previously established methods with minor modifications [18]. Testing for seasonal influenza (A (H1N1), A (H3N2) and B) was performed using the Artus® Influenza LC RT-PCR kit (Qiagen®) with the Light Cycler 2.0 (Roche®) using established methods [19], [20]. Testing was performed nonselectively on all available swabs.

### Statistical analysis

Results for patients with CAP who tested positive for influenza A 2009 (H1N1) were compared with other CAP patients. Testing for statistical significance of numerical data was conducted using the Mann Whitney U test while categorical data was examined using Fisher's exact test. In cases with more than two categories a Pearson Chi square test was applied (SPSS Statistics 19). Results are displayed as mean values with 95% confidence intervals (CI) or percentages. Odds ratio was calculated and presented with the 95% CI. Results were considered statistically significant when a two tailed *P* value was <.05.

## Results

### Patient recruitment

During the 12 month study period a total of 397 patients were admitted due to pneumonia. Of these, 9 were discharged without notification to the investigators and 15 suspected CAP patients declined participation. Of the remaining 373 patients, 60 had exclusion criteria [15]. The remaining 313 patients constitute the study cohort of patients with CAP requiring hospitalization, representing 93% of eligible patients. No patients were lost to follow-up.

### Influenza epidemiology in the community

**Figure 1:**
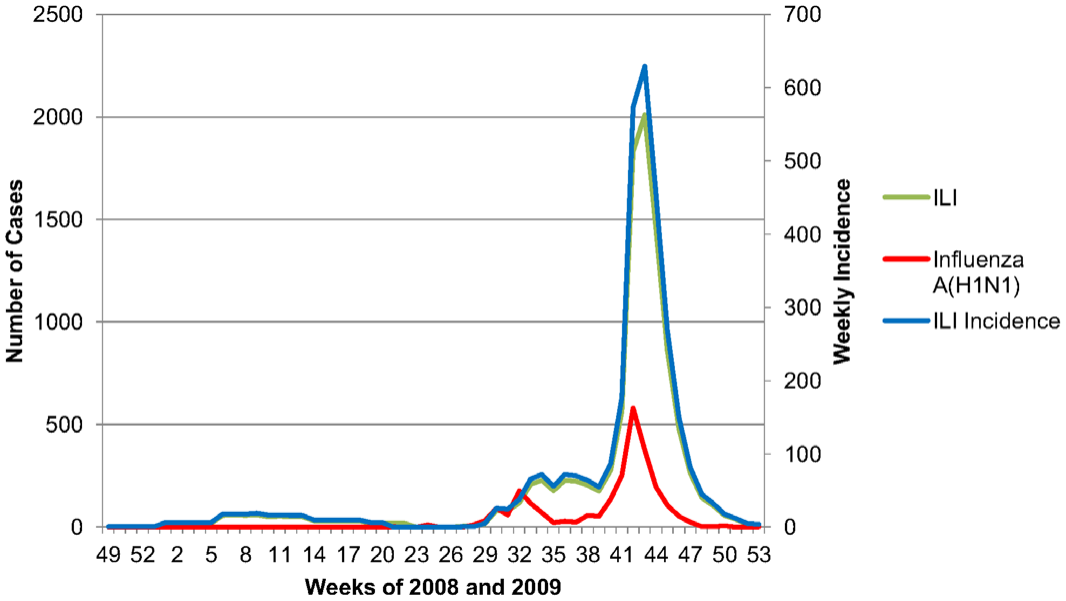
Epidemiology of influenza in Iceland, December 2008-December 2009, showing the number of reported cases of influenza-like illness (ILI) and confirmed influenza A 2009 (H1N1) (left y-axis) and weekly ILI incidence per 100 000 population (right y-axis). In Iceland approximately 62% of all virologically confirmed cases and ILI were in Reykjavik [21]. (Ref: http://www.influensa.is/pages/1505).

Influenza activity during the 2008–2009 influenza season was low in Iceland. The first cases of influenza A 2009 (H1N1) in the country were diagnosed in late May, but the activity remained low until fall. Overall, 9887 individuals were reported with influenza-like illness (ILI) from week 27 until week 52 in 2009. This constitutes an incidence of 6194 cases per 100 000 inhabitants per year [21]. The vast majority of these cases were diagnosed from June 29^th^–November 6^th^ 2009 (8650 of 9987 total cases) [21]. These findings are summarized in figure 1.

### Influenza and community-acquired pneumonia in the hospital

Prior to the pandemic, two CAP patients were diagnosed with seasonal H3N2 influenza pneumonia. The first patient admissions with influenza A 2009 (H1N1) were in August and reached a peak in October, synchronous with ILI activity in the society at large. A total of 114 adult patients with confirmed 2009 H1N1 infection were admitted to our centre, and 22 (19%) of those patients had infiltrates on chest X-ray and thus were included in the study. During its peak, influenza 2009 (H1N1) pneumonia accounted for 38% of all admissions for CAP.

### Microbiology

**Figure 2:**
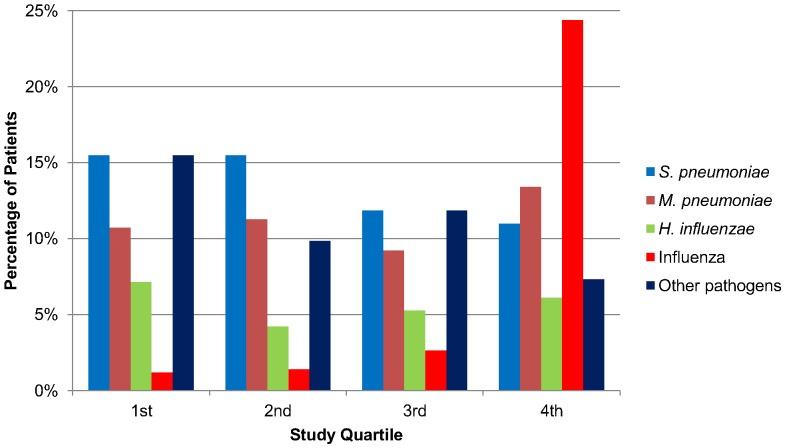
Etiologic causes of community acquired pneumonia (CAP) identified during the 12-month study, by quarters. The proportion of total pneumonia admissions accounted for by each etiology for each quartile is shown. Influenza during the first and second quartiles was caused by seasonal influenza H3N2 whereas all influenza cases during the third and fourth quartiles were pandemic influenza (H1N1). Less frequently encountered pathogens listed as “other” included *M. catarrhalis*, *S. aureus*, *C. pneumoniae*, *Legionella* species, *P. aeruginosa* as well as various streptococcal species.

In total, 139 of 313 patients received 154 etiologic diagnoses, thus giving a diagnostic yield of 44.4% for the overall cohort. *S. pneumoniae* was the most common pathogen, found in 30% of diagnosed cases. During the study period no major shift in the prevalence of pathogens other than influenza was noted (figure 2). Bacterial co-pathogens were found in three 2009 (H1N1) CAP patients (14%). One patient had a positive *S. pneumoniae* urinary antigen test, and one had both *S. pneumoniae* and *S. aureus* cultured from high-quality sputum. In addition *Burkholderia pseudomallei* was cultured from blood of a traveler returning from Thailand. By including patients with positive cultures from lower-quality respiratory specimens, co-infections increase to five (23%).

### Clinical characteristics of patients with influenza CAP compared to other CAP patients

The clinical characteristics of patients with CAP due to influenza A 2009 (H1N1) and patients with other causes for CAP are compared in table 1. The influenza patients were younger (*P*<.001) and had a lower prevalence of chronic disease (*P* = .01). Further, they were more likely to report hemoptysis and dyspnea, and had lower platelet and white blood cell counts than patients with CAP due to other etiologies (table 2). A significant difference in chest X-ray appearance was found, with a bilateral interstitial infiltrate being strongly associated with the 2009 pandemic strain.

**Table 1:** Comparison of CAP Patients by Etiology – Characteristics and Underlying Conditions.

Characteristics	2009 (H1N1) Influenza CAP (n = 22)	CAP, other Etiology n = 291	Odds ratio (95% CI)	*P* value^a^
Age, mean (95% CI), y	44.0 (37.1–50.9)	64.4 (62.1–66.7)		**<.001**
Male, No. (%)	13 (59)	141 (49)	.65 (.27–1.57)	.38
Current smokers, No. (%)	8 (36)	57 (20)	2.35 (.94–5.86)	.10
Alcohol abuse, No. (%)	2 (9)	18 (6)	1.52 (.33–7.00)	.64
Immune suppression^b^, No.(%)	0 (0)	25 (9)	.91 (.88–.95)	.24
**Medication use at admission, No. (%)**				
Corticosteroids	1 (5)	28 (10)	.45 (.06–3.45)	.71
PPI	3 (14)	83 (29)	.40 (.11–1.37)	.21
Statins	3 (14)	65 (22)	.55 (.16–1.91)	.43
Antibiotics	10 (45)	97 (33)	2.0 (.84–4.8)	.16
**Underlying conditions, No. (%)**				
COPD	2 (9)	81 (28)	.26 (.06–1.13)	.08
Asthma	3 (14)	41 (14)	.96 (.27–3.40)	.99
DM I	0 (0)	3 (1)	.99 (.98–1.00)	.99
DM II	1 (5)	38 (13)	.32 (.04–2.43)	.33
Ischemic heart disease	1 (5)	63 (22)	.17 (.023–1.31)	.06
Heart failure	2 (9)	35 (12)	.73 (.16–3.26)	.99
Cerebrovascular disease	0 (0)	18 (6)	.94 (.91–.97)	.63
Renal failure	1 (5)	31 (11)	.40 (.05–3.07)	.71
Liver disease	1 (5)	5 (2)	2.72 (.30–24.39)	.36
Malignancy	0 (0)	9 (3)	.97 (.95–.99)	.99
Any chronic disease^c^	7 (32)	172 (59)	.32 (.13–.80)	**.01**

CAP, community acquired pneumonia; CI, confidence interval; PPI, proton pump inhibitor; COPD, chronic obstructive pulmonary disease; DM, diabetes mellitus.

a *P* values<.05 shown in bold.

b Immune suppression due to medications or malignancy.

c Any chronic disease is a composite of the conditions listed above.

**Table 2:** Comparison of CAP Patients by Etiology – Symptoms, Test Results and Severity Scores.

Characteristics	2009 (H1N1) Influenza CAP (n = 22)	CAP, other Etiology (n = 291)	Odds ratio (95% CI)	*P* value^a^
**Self-Reported Symptoms, No. (%)**				
Cough	20 (90)	229 (79)	2.71 (.62–11.90)	.27
Fever	21 (95)	243 (84)	4.15 (.55–31.58)	.22
Sputum production	12 (55)	150 (52)	1.13 (.47–2.96)	.83
Hemoptysis	6 (27)	28 (10)	3.52 (1.28–9.73)	**.02**
Dyspnea	21 (95)	199 (68)	9.71 (1.29–73.28)	**.01**
Headache	12 (55)	95 (33)	2.48 (1.03–5.94)	.06
Abdominal pain	4 (18)	40 (14)	1.39 (.45–4.33)	.53
Chest pain	9 (41)	132 (45)	.83 (.35–2.01)	.83
Diaphoresis	12 (55)	83 (29)	2.37 (.98–5.72)	.06
Chills	14 (64)	159 (55)	1.45 (.59–3.57)	.51
Diarrhea	7 (32)	43 (15)	2.69 (1.04–6.99)	.06
**Vital signs on admission, mean (95% CI)**				
Temperature, °C	38.4 (38.0–38.7)	38.1 (38.0–38.2)		.17
Heart rate, min^−1^	102 (93–111)	97 (95–100)		.38
Systolic BP, mm Hg	128 (119–136)	130 (127–133)		.83
Diastolic BP, mm Hg	70 (66–74)	69 (67–70)		.54
MAP, mm Hg	89 (85–94)	89 (87–91)		.68
RR, min^−1^	25 (21–29)	24 (23–25)		.66
SpO2, %	93 (91–95)	92 (91.5–93)		.96
SpO2 worst value^b^, %	90 (86–94)	92 (91–92)		.33
**Blood test results, mean (95% CI)**				
WBC count, 10^3^/µL	7.9 (5.4–10.4)	12.8 (12.1–13.5)		**<.001**
WBC count, worst value, 10^3^/µL	7.8 (5.2–10.3)	13.2 (12.5–13.9)		**<.001**
Hemoglobin, g/dL	13.9 (13.1–14.6)	12.9 (12.7–13.1)		**.01**
Hematocrit, %	39.7 (37.8–41.6)	37.9 (37.4–38.5)		**.03**
Hematocrit, worst value, %	38.1 (36.0–40.3)	36.3 (35.7–36.9)		**<.05**
Platelet count, ×10^3^/µL	208 (166–250)	252 (239–265)		**.01**
Sodium, mEq/L	137 (136–138)	139 (138–139)		**.004**
Sodium worst value, mEq/L	137 (136–138)	139 (138–139)		**.01**
Potassium, mEq/L	3.9 (3.7–4.1)	4.0 (3.97–4.1)		.12
Potassium worst value, mEq/L	3.9 (3.7–4.1)	4.0 (3.9–4.1)		.18
Urea, mg/dL	14.6 (10.9–18.5)	20.5 (18.5–22.4)		**.04**
Glucose, mg/dL	110 (97–123)	132 (124–141)		.09
CRP, mg/L	120 (83–157)	133 (121–144)		.71
**Radiological results, No. (%)**				
Lobar infiltrate	7 (32)	202 (69%)	.21 (.08–.52)	**.001**
Bilateral interstitial infiltrate	11 (50)	27 (9)	9.78 (3.88–24.65)	**<.001**
Other appearance	4 (18)	62 (22)	.82 (.27–2.51)	.99
**Severity scores, mean (95% CI)**				
PSI score	2.05 (1.60–2.49)	2.87 (2.73–3.01)		**<.001**
CURB-65 score	1.23 (.96–1.50)	1.61 (1.52–1.70)		**.02**
APACHE II score	7.41 (5.64–9.18)	9.38 (8.78–9.99)		.09

CAP, community acquired pneumonia; CI, confidence interval; BP, blood pressure; MAP, mean arterial pressure; RR, respiratory rate; SpO2, pulse-oximetry; WBC, white blood cell; CRP, C-reactive protein; PSI, pneumonia severity index.

a P-values<.05 shown in bold.

b Worst value denotes the worst noted value during the first 24 hours of admission.

### Pneumonia Severity scores

All patients received PSI, CURB-65 and APACHE II scores. Patients with influenza A 2009 (H1N1) CAP had a significantly lower PSI and CURB-65 scores on admission than other patients with CAP (table 2). When the CURB-65 score was recalculated by omitting the age criteria (one point for age over 65), the difference between the two patient groups became non-significant (1.14 vs. 1.20). The PSI risk class is derived from various clinical parameters which give points, including one point for each year of age for men, and age in years −10 for women [5]. The difference in mean age between the two groups (44.0 [37.1–50.9] vs. 64.4 [62.1–66.7]) corresponded roughly to the difference in mean PSI values (56.3 [43.8–68.7] vs. 79.2 [75.2–83.2]).

### Treatment, length of stay and outcomes

All admitted patients received intravenous antibiotic therapy. In the influenza group 86% received treatment with oseltamivir (table 3). Influenza CAP patients more commonly received coverage for atypical bacterial agents than other patients with CAP. Patients with influenza pneumonia displayed a non-significant trend towards a longer hospital stay and longer duration of antimicrobial treatment. They also received a higher level of care, with 41% being admitted to intensive care unit (ICU) as compared with 6% of other CAP cases (*P*<.001) and 14% requiring invasive ventilation as compared with 2% of other CAP cases (*P*<.001). Influenza CAP patients admitted to ICU had worse oxygen saturation levels than other influenza patients with the mean worst SpO2 saturation of the groups during their first 24 hours of admission being 84% vs. 94% (P = .005). The values of C-reactive protein (CRP) differed significantly; only three of 13 non-ICU patients had a CRP of over 100 mg/L, and two of these had documented bacterial co-infections. In contrast, seven of nine ICU patients had CRP over 100 mg/L. No fatalities occurred in the influenza group compared with a mortality of 3% (n = 10) in the non-influenza group.

**Table 3:** Comparison of CAP Patients by Etiology – Treatment and Outcome.

	2009 (H1N1) Influenza CAP (n = 22)	CAP, other Etiology (n = 291)	Odds ratio (95% CI)	*P* value^a^
**Therapy, No. (%)**				
IV abx therapy	22 (100)	291 (100)		
oseltamivir	19 (86)			
Atypical coverage^b^	15 (68)	111 (38)	3.53 (1.39–8.92)	**.01**
Duration of abx, mean (CI), days	14.3 (8.5–20.2)	11.5 (11.0–11.9)		.75
Length of stay, mean (CI),days	9.6 (6.2–13.0)	7.4 (6.8–7.9)		.13
ICU admission	9 (41)	16 (5)	11.7 (4.4–31.5)	**<.001**
Invasive ventilation	3 (14)	5 (2)	9.03 (2.01–40.67)	**.01**
In-hospital mortality	0 (0)	10 (3)	.96 (.94–.99)	
**Etiologic testing, No (%)**				
Sputum acquired	10 (45)	147 (51)	.83 (.35–1.98)	.83
Representive sputum acquired	5 (23)	97 (33)	.60 (.21–1.67)	.48
Blood culture	22 (100)	211 (73)	.73 (.68–.78)	**.002**
BAL	1 (5)	8 (3)	1.69 (.20–14.11)	.49
UAT *S. pneumoniae*	15 (68)	210 (72)	.83 (.33–2.10)	.81
UAT *L. pneumophila*	13 (59)	197 (68)	.69 (.29–1.67)	.48

CAP, community acquired pneumonia; CI, confidence interval; IV, intravenous; abx, antibiotic; UAT, urine antigen test;; ICU, intensive care unit; BAL, bronchoalveolar lavage.

a P-values<.05 shown in bold.

b Atypical coverage denotes empiric antimicrobial treatment including coverage for “atypical” bacterial organisms.

## Discussion

Here, we present the results of a prospective population-based study of influenza in the context of pneumonia, a serious clinical presentation of pandemic influenza. We are not aware of any prospective studies comparing clinical characteristics of patients admitted with 2009 H1N1 influenza pneumonia with those of CAP caused by other pathogens. During the height of the pandemic in Iceland, 38% of patients admitted with CAP tested positive for H1N1. Almost one in five (19%) admitted patients with confirmed influenza had concurrent pneumonia. This is higher than figures from Argentina (11%) and Beijing (12%), and similar to Mexico City (18%), while much higher figures were reported from California (66%) and national sampling from the United States (43–46%) [13], [22], [23], [24], [25], [26]. It is important to note the extremely variable methodology and setting of these studies which might explain the different results. The admission rate of 41 per 100 000 inhabitants in our study was similar to figures from the US, where rates of 38 per 100 000 inhabitants were noted during the peak of the pandemic [27].

Interestingly, hospital admissions for CAP caused by agents other than influenza were similar to or below the study period's monthly average for three of the four months of peak ILI activity (data not shown). Therefore, the epidemic in the community did not seem to lead to any discernible increase in bacterial pneumonia requiring admission (See figure S1). It is important to note that preventive measures, such as mass vaccination, initiated in mid-October, and antiviral treatment were being enforced simultaneously. Two weeks after conclusion of our study 24% of the population had been vaccinated according to official figures.

The timing of the study provided a unique opportunity to compare patients with CAP due to pandemic influenza A 2009 (H1N1) to those with CAP caused by other agents. Our results demonstrate that pneumonia caused by the novel pandemic strain was more severe than CAP of other microbial etiology, despite the fact that these were younger patients with less co-morbidity than other CAP patients. Patients with CAP due to influenza A 2009 (H1N1) were significantly more likely to require ICU admission and receive invasive ventilation. Previous studies from tertiary care hospitals have indicated a more severe course of illness and a higher mortality rate [28], which might be explained by selection bias. However, our prospective population-based study is in agreement with those results.

As a group, patients with CAP due to pandemic influenza A 2009 (H1N1) were more symptomatic than other CAP patients. Interestingly one-third of influenza pneumonia patients reported hemoptysis, which corresponds to the descriptions of the initial patients in Mexico, but is rarely encountered in CAP from other etiologies [24], [29]. A bilateral interstitial infiltrate on a chest X-ray was characteristic but one third of the influenza patients had a lobar infiltrate, similar to previous descriptions [30].

The prevalence and importance of bacterial co-infections with *S. pneumoniae* and *S. aureus* in patients with influenza is debated [2]. Our results demonstrate unequivocal co-infections in only three patients (14%). Historical reports and some more recent studies have indicated a much higher rate [31], [32]. Antibiotics prior to admission might give a partial explanation; 11 of 22 patients reported having received antibiotics and none of the co-infected patients was in this group. Even when lower-quality specimens were included the rate of co-infection was 23%. However, if patients with previous antibiotic exposure are excluded, and those with positive lower-quality respiratory specimens are included, the proportion of bacterial co-infections reaches 45%. Some studies have shown higher levels of suspected co-infection, but they often rely on upper airway samples which are not adequate to diagnose a lower respiratory tract infection with pathogens such as *S. pneumoniae* [33].

PSI and CURB-65 are two major scoring systems with similar validity, designed to risk-stratify patients presenting with CAP. Both scoring systems are recommended for routine use by most major published pneumonia management guidelines, including the Infectious Diseases Society of America, American Thoracic Society and the British Thoracic Society [6], [7]. Although originally designed to designate patients suitable for out-patient treatment, these scoring systems have also been used to help stratify inpatients according to severity with such recommendations entering some national guidelines [34]. Despite having a more severe disease the 2009 (H1N1) CAP patients had, paradoxically, lower PSI and CURB-65 scores than other CAP patients. In addition, neither score predicted the need for ICU admissions or mechanical ventilation among the influenza patients. This discrepancy seems to be explained by points given for age. This has been previously noted for CURB-65, but the results were based on retrospective analysis of selected cases from a referral centre and thus prone to selection and referral biases [35]. The failures of both scoring systems points out weaknesses in the current methods to stratify patients with CAP. Although increasing age is traditionally associated with greater severity and worse prognosis for most illnesses and thus independently increases severity scores such as PSI, APACHE II and SAPS II, this pandemic proves to be an exception. It is plausible that given the higher prevalence of cross-reactive antibodies in the population above the age of 60, increasing age was relatively protective against severe illness [36]. Importantly, the PSI and CURB-65 scoring systems were developed decades after the last influenza pandemic and not intended for use during an epidemic with a novel viral agent [5]. Our results underscore the importance of clinical judgment in decision-making, as the average PSI and CURB-65 scores for admitted patients were below criteria recommending admission to hospital [4], [5]. Therefore, we feel that neither of these scores in their present form should be used for clinical decision-making during epidemics in populations with low herd immunity. New or amended scoring systems with less focus on age might prove to be more robust under these conditions.

While most demographic data in our study corresponds to previously published results we had no mortality in our group. There were two deaths in the country attributed to the pandemic, neither of which fulfilled the study criteria for CAP [21]. Even by including these patients the mortality for inpatients (1.5%) was substantially lower than reported from Beijing (14%), Mexico City (39%) and the United States (4.6%) [24], [25], [27]. The difference may be related to sample size, inclusion criteria, differences in patient host factors, pneumococcal carriage in the population, or the level of care in these studies [37].

The major strength of this study is the prospective population-based design, with high inclusion rate of consecutive patients, thus providing high external validity. All available samples were screened for influenza which provides detailed information regarding the impact of the epidemic. However, it has limitations. Despite including 94% of available patients the number of cases is low. It is possible that the proportion of CAP attributable to influenza may be underrated due to false negative test results from PCR of nasopharyngeal swabs. However, no increase in undiagnosed or bacterial pneumonia was noted during the epidemic period suggesting that this should was not a major problem. Broncho-alveolar lavage may be more sensitive in the diagnosis of influenza pneumonia [35], but subjecting all admitted patients with CAP to bronchoscopy is not ethically acceptable. Furthermore, occasional patients may have been admitted to smaller hospitals. This is unlikely to make an impact in our results however, since our hospitals hold 70% of all hospital beds in the country.

In summary, patients with pneumonia due to influenza A 2009 (H1N1) were younger than other patients with CAP, and required intensive care and mechanical ventilation more frequently. Despite having a more severe disease, they had lower PSI and CURB-65 scores on admission, suggesting that modification of these prediction rules may be warranted in the setting of novel pathogens to which the herd immunity is low. Last, but not least these results remind us of the importance of clinical acumen in decision making.

## Supporting Information

Figure S1 Total number of patients admitted with community-acquired pneumonia (CAP) and influenza CAP, by study quarters.
(TIF)
