# Supplementary figures and images for: Severity of Influenza A 2009 (H1N1) Pneumonia Is Underestimated by Routine Prediction Rules. Results from a Prospective, Population-Based Study
Source: PLoS One. 2012 Oct 11;7(10):e46816. doi: 10.1371/journal.pone.0046816 (PMC3469650; doi:10.1371/journal.pone.0046816)

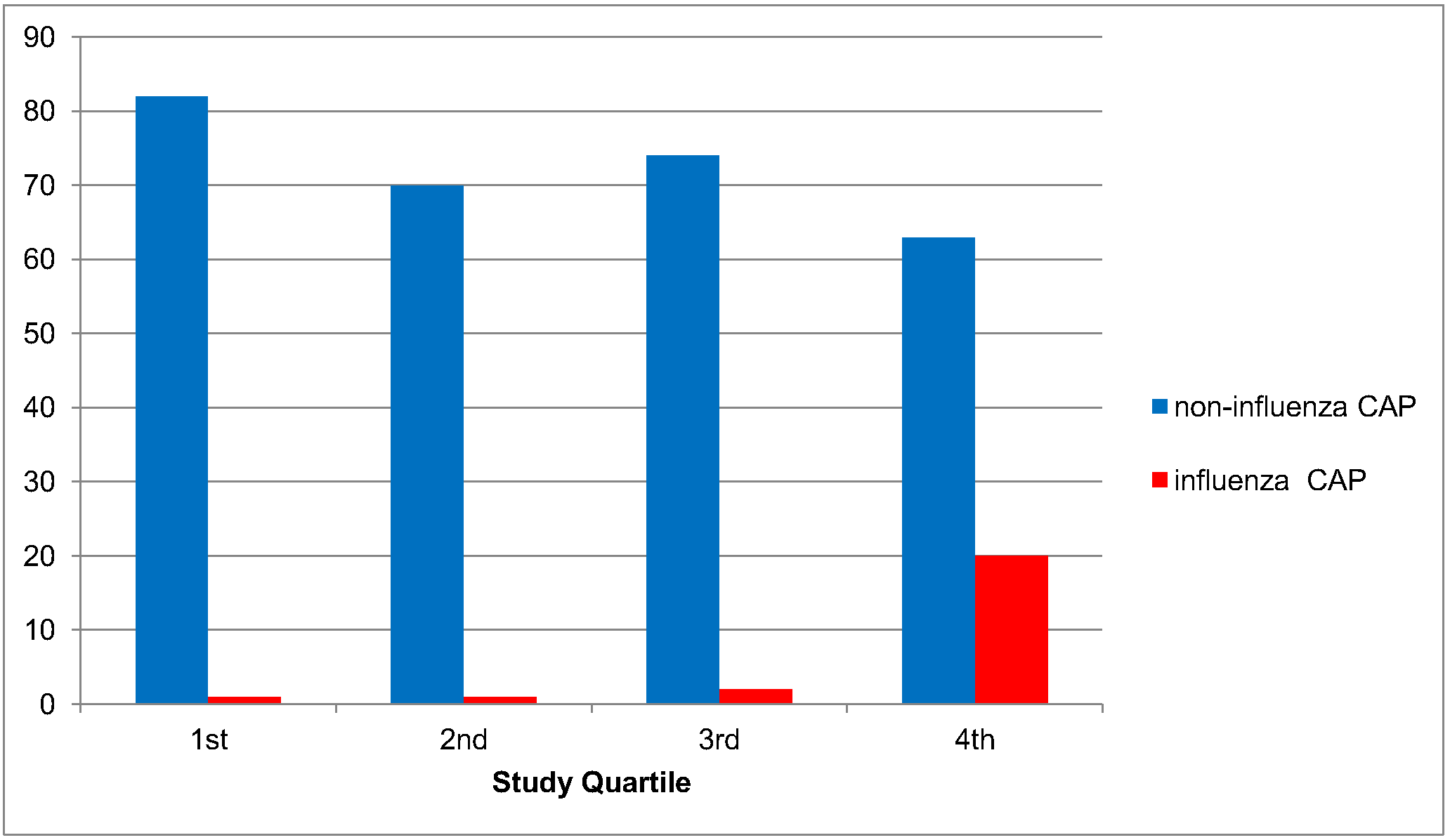

Supplement: Figure S1 — Total number of patients admitted with community-acquired pneumonia (CAP) and influenza CAP, by study quarters. (TIF) [file pone.0046816.s001.tif]
